# Role of Heat Shock Proteins in Glaucoma

**DOI:** 10.3390/ijms20205160

**Published:** 2019-10-18

**Authors:** Teresa Tsai, Pia Grotegut, Sabrina Reinehr, Stephanie C. Joachim

**Affiliations:** Experimental Eye Research, University Eye Hospital, Ruhr-University Bochum, Bochum, In der Schornau 23-25, 44892 Bochum, Germany; teresa.tsai@rub.de (T.T.); pia.grotegut@rub.de (P.G.); sabrina.reinehr@rub.de (S.R.)

**Keywords:** glaucoma, heat shock protein, HSP27, HSP60, optic nerve, retina

## Abstract

Glaucoma, one of the most common causes of blindness worldwide, is a multifactorial neurodegenerative disease characterized by damage of retinal ganglion cells and optic nerve degeneration. However, the exact mechanism leading to glaucoma is still not understood. Evidences suggest an immunological involvement in the pathogenesis. Among other immune responses, altered autoantibody patterns were found in glaucoma patients. Especially elevated antibody levels against heat shock proteins (HSPs), like HSP27 or HSP60, were identified. In an animal model, an immunization with these HSPs induced a pressure-independent retinal ganglion cell degeneration and axon loss, hence mimicking glaucoma-like damage. In addition, development of autoreactive antibodies, as well as a glia and T-cell activation, were described in these animals. Recently, we noted that intravitreal HSP27 injection likewise led to a degeneration of retinal ganglion cells and their axons. Therefore, HSP27 might have a direct damaging effect on retinal cells, and might play a key role in glaucoma.

## 1. Introduction

Heat shock proteins (HSPs) are evolutionarily highly conserved polypeptides. They are originally identified as stress proteins against thermal stress, and occur under physiological conditions as molecular chaperones and can have anti-apoptotic activities [1,2]. Interestingly, some HSPs are constitutively expressed intracellularly, whereas the expression of others is induced by internal or external stimuli, like cell cycle alterations, heat, inflammation, oxidative stress, or toxic substances [2]. Based on their molecular weight, HSPs are subdivided into several families, such as small HSPs (12–43 kD) as well as HSP40, HSP60, HSP70/110, HSP90, and HSP100 [3]. Due to their size, each family has a specific physiological function and location within the cell [4]. The family of small HSPs consists of eleven members, including HSP27, as well as α-, β-, and γ-crystallin [3]. Under physiological conditions, these proteins are constitutively synthesized in relatively small amounts, but the expression is highly up-regulated after stress exposure [5]. Predominantly, small HSPs are involved in stress reactions, apoptosis prevention, cytoskeleton stabilization, and protein folding [4,6]. The HSP60 family members are mainly mitochondrially expressed chaperones that can be translocated into the cytosol in stress situations, to be later transported to the cell surface and released into the environment [7,8,9].

This review deals with the interaction between HSPs and the immune system in glaucoma. The focus lies on the importance of the interaction for the glaucoma patient, as well as the detailed investigation of the HSP mechanisms in corresponding animal models.

## 2. Interaction of Heat Shock Proteins and the Immune System

**Figure 1:**
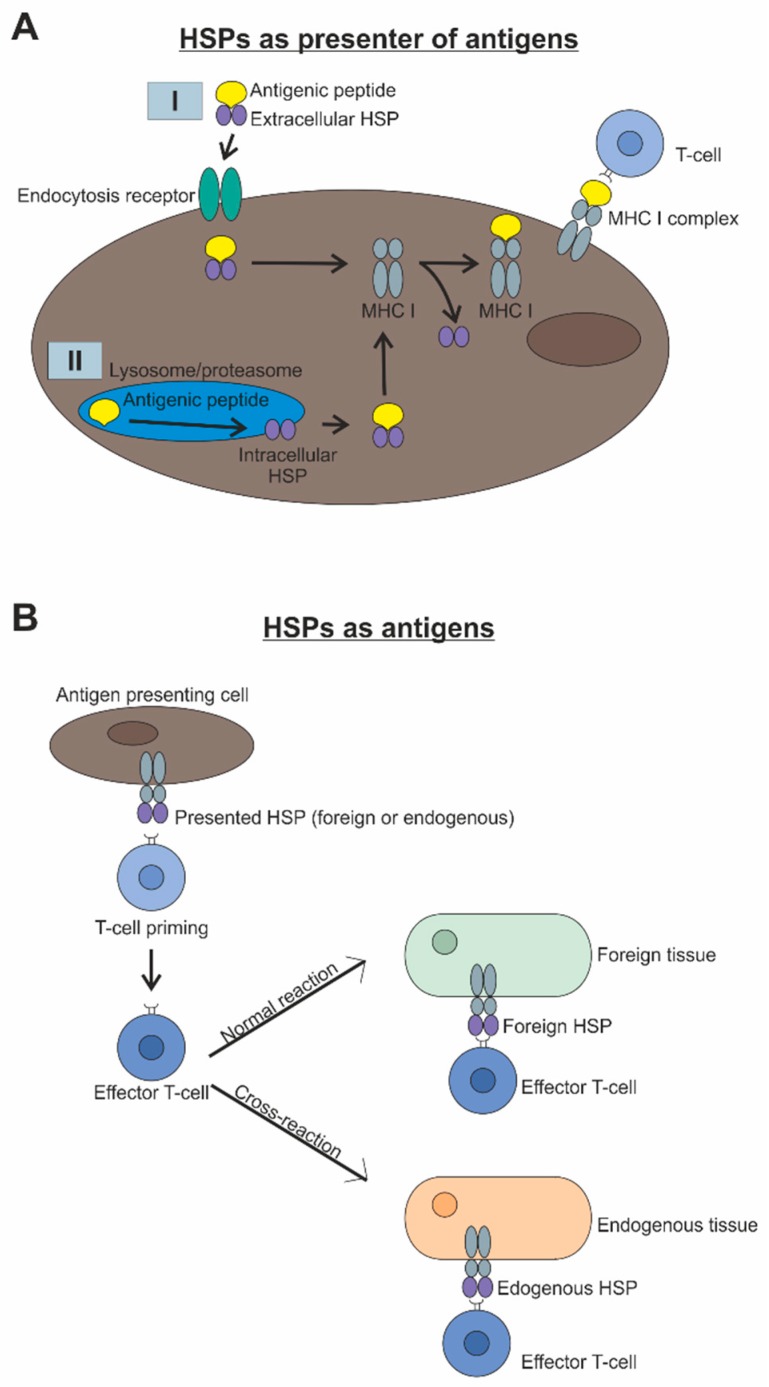
(**A**) HSPs can bind antigenic peptides either extracellularly (I) or intracellularly (II). Subsequently, the HSP-antigen complex reaches the endoplasmic reticulum, where the loading of the major histocompatibility complex (MHC) occurs. Finally, the MHC-antigen complex is transported to the cell membrane, where the antigen is presented to the T-cell. (**B**) HSPs as antigens are presented by antigen presenting cells to T-cells. Afterwards, primed T-cells become effector cells. Subsequently, these effector cells normally recognize foreign HSPs on the surface of foreign tissue and induce determination of it. However, when T-cells are primed by endogenous HSPs, which are normally used for chaperone or anti-apoptotic activities, these T-cells can cross-react and destroy endogenous tissue.

As mentioned before, HSPs have a variety of functions, including the stimulation of both the innate and adaptive immune system [10,11]. In the course of this, they take up the role of antigen presentation. Immunogenic peptides are either extracellularly bound by HSPs, e.g., HSP70, and taken into the cell via endocytosis, or they are bound intracellularly into lysosomes or proteasomes, by e.g., HSP70 or HSP90, after degradation [10,11,12]. Subsequently, the antigen transport to the endoplasmic reticulum takes place, where the loading of the major histocompatibility complex (MHC) I occurs. Finally, the MHCI-antigen-complexes are transported to the cell membrane, where the antigen is presented to the T-cell (Figure 1A). Afterwards, primed T-cells become effector cells. The subsequent immune response depends on whether the presented antigens are foreign or endogenous [10,11,13]. If the antigens are foreign, a specific immune response takes place and induces determination of the foreign tissue. In contrast, undesired presentation of endogenous HSPs, which normally work as e.g., chaperones, leads to a cross-reaction and devastation of the endogenous tissue (Figure 1B).

In addition to their antigen-presenting function and as mentioned before, HSPs themselves can be recognized as antigens. It has been known for a long time that HSP60 is a common antigen for gram-negative bacteria and hence a major target of the immune defense in infections [14,15]. Consequently, HSP60 represents a danger signal to the innate immune system and therefore a protective mechanism for the human organism. However, in addition to this positive effect, HSPs as antigens pose a potential risk, particularly based on their evolutionarily conserved structure [10]. Due to this, T-cells originally targeted against the bacterial HSP may cross-react with homologous self-peptides and lead to autoimmune damage (Figure 1B). Such cross-reactive T-cells have already been localized in serum samples of patients with rheumatoid arthritis [16,17] and play an important role in various autoimmune diseases, like diabetes mellitus and atherosclerosis [18,19,20,21]. While interactions between bacterial or self-HSP and components of the host’s immune system lead to stimulation of the humoral (auto)-immune response and the production of (auto)-antibodies to HSP, there are also studies indicating immunosuppressive activity of some HSPs by interacting with anti-inflammatory regulatory T-cells [22,23]. Therefore, the diversity of a HSP reaction within the immune response has to be further investigated. However, pathogens, such as bacteria, are not the only trigger to induce tissue and cell damage, as well as an activation of the immune system. Trauma from mechanical forces, excessive heat, chemical attack, radiation, or depletion of oxygen and nutrients might also have these effects [24].

In summary, these data show that HSPs have a major impact on the immune system, and therefore also play an important role in the development of numerous autoimmune diseases, such as multiple sclerosis [25]. But HSPs are also candidates of interest in the pathogenesis of neurodegenerative conditions, such as Alzheimer’s disease [26] or glaucoma [27].

## 3. Role of Heat Shock Proteins in Glaucoma Patients

Glaucoma is a multifactorial, neurodegenerative disease, defined as a neuropathy with changes at the optic nerve head and progressive retinal ganglion cell loss. It is one of the most common causes of irreversible vision loss worldwide [28]. However, the exact mechanism leading to glaucoma is still not understood. Hence, current research approaches aim to decode these pathomechanisms. Numerous studies in recent years have addressed alterations of the cellular and humoral acquired immune response in glaucoma patients. Wax et al. were able to detect abnormal autoantibodies and paraproteins in normal pressure glaucoma patients more than 20 years ago [29]. Since an immunological component is considered to play a role in the pathogenesis of glaucoma, possible antibody changes in glaucoma patients were investigated more closely. The group of Wax was able to demonstrate an increased serum immunoreactivity against the HSPs αA- and αB-crystallin, HSP27, and HSP60 in a patient with normal pressure glaucoma [30]. Back then, the authors already postulated that those autoantibodies could be involved in neuronal cell death. In the same year, this research group also investigated serum samples from normal as well as high pressure glaucoma patients (primary open-angle glaucoma, POAG) and healthy control subjects. Both patient groups exhibited an increased autoantibody titer against small HSPs, including α- and β-crystallin and HSP27 [27]. Quite similar observations were made when comparing study populations in Japan and the USA. Both populations showed elevated antibody levels against small HSPs in glaucoma patients, which were higher in normal pressure ones [31]. 

By applying antibodies against crystallin or HSP27 to postmortem human retinal tissue or immortalized retinal rat cells, apoptosis could be induced. Hence, these antibodies might be taken up by cells and induce cell death [27]. Also, HSP27 antibodies were internalized by cells, when applied to human retinal tissue, leading to a subsequent cell death [32]. An increased immunoreactivity against anti-HSP27 and anti-HSP60 antibodies could be observed in human donor retinae from normal and high pressure glaucoma patients [33]. Later studies analyzed not only a few specific antibody responses in patient samples, but investigated complex antibody patterns. They found multiple alterations of the autoantibody pattern in serum samples of patients with normal and high pressure glaucoma [34]. Interestingly, autoantibodies against certain HSPs, like αB-crystallin or HSP70, were also up-regulated in aqueous humor samples from normal pressure glaucoma patients [35]. Boehm et al. [36] compared autoantibodies in the aqueous humor and serum samples from the same POAG patients using microarrays. They noted elevated HSP70 antibody levels in serum samples, while βL-crystallin was down-regulated. Data from aqueous humor specimens indicated increased levels of HSP10 and αB-crystallin in the glaucoma group. A strong correlation was observed in serum and corresponding aqueous humor samples in regard to the antibody scores [36]. Moreover, not only normal and high-pressure glaucoma patients exhibited altered autoantibody titers. In another study, high antibody levels against HSP27 were identified in the aqueous humor from POAG and pseudoexfoliation glaucoma patients. In this study, significantly different antibody patterns were noted between POAG and controls, as well as pseudoexfoliation glaucoma and controls, but not between the two glaucoma groups [37]. Saccà et al. analyzed aqueous humor samples from an Italian study population using antibody arrays. They noted increased titers of HSP60 and HSP90 in POAG patients [38]. A gene polymorphism for HSP70 was also observed in POAG patients from Poland, Japan, and Pakistan [39,40,41]. In addition, antibody levels to HSP60Sp, Streptococcus pyogenes HSP60, were elevated in POAG patients from Mexico [42]. 

These findings indicate that autoantibodies against HSPs in glaucoma patients could be directly involved in disease onset and progression. While several studies found elevated levels of antibodies against HSPs in glaucomatous serum or aqueous humor samples, some studies came to another conclusion. Grabska-Liberek et al. did not note any differences in serum levels of antibodies against HSP60 in their glaucoma patients [43]. In addition, Nowak et al. observed similar expression levels of antibodies against HSP70 in serum samples from POAG and controls [44]. Nevertheless, this putative disagreement to other studies noticing differences in expression patterns of HSP antibodies could be explained by different glaucoma subtypes analyzed or study populations. 

Multiple studies suggest an autoimmune component of glaucoma. However, it remains unclear whether HSP antibodies in the sense of an auto-aggressive response cause direct damage to the retinal ganglion cells or are just an epiphenomenon. To further elucidate the detailed role of HSPs and its autoantibodies, several studies have been conducted in different glaucoma animal models.

## 4. Heat Shock Proteins Induce Glaucoma-Like Damage in Animal Models 

To find out if increased HSP antibody levels play a role in disease development or are just a secondary response, many studies were carried out. To investigate the role of HSPs in glaucoma, Wax et al. immunized rats with HSP27 or HSP60, and were able to detect a significantly lower density of retinal ganglion cells after 28 days. The most pronounced cell loss took place in the central area of the retina. The authors therefore concluded that ganglion cell loss in this animal model reflects the onset of normal pressure glaucoma patients. In addition, a significantly higher number of αβ receptor-positive T-cells were detected in retinal flatmounts of these animals 14 and 21 days after immunization with HSPs. These cells were no longer detectable at later points in time [45]. 

**Figure 2:**
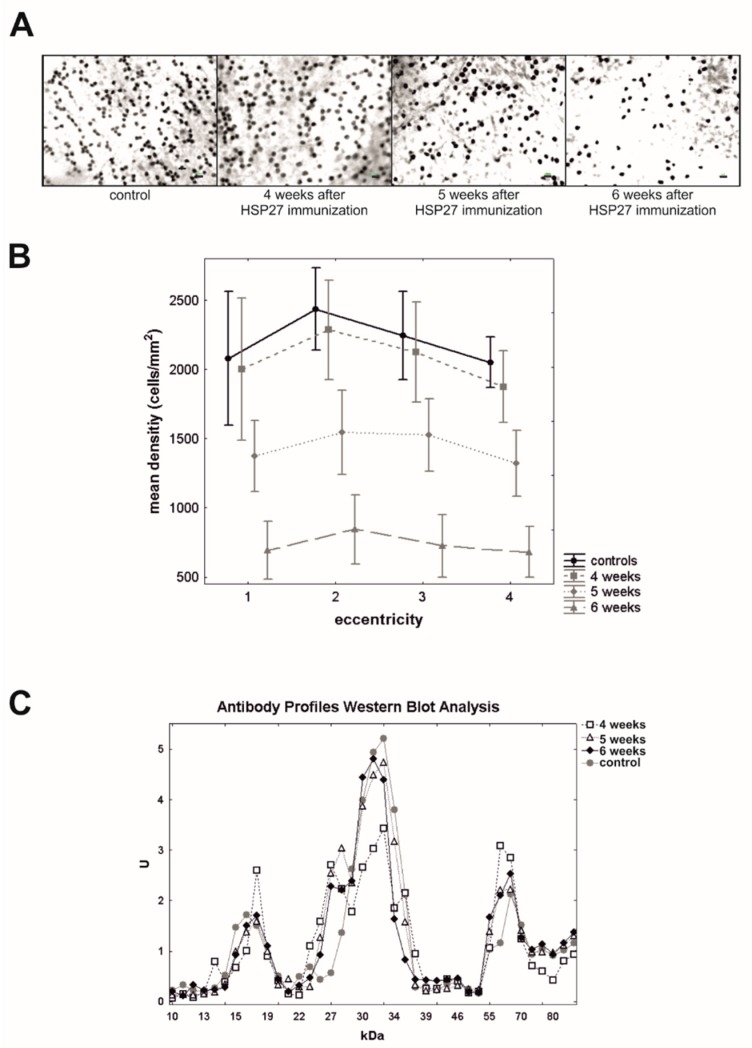
(**A**) Exemplary retinal flatmounts labeled with Brn3a from control rats and animals, which were immunized with HSP27 after four, five, and six weeks. (**B**) Mean retinal ganglion cell density of immunized rats after four, five, and six weeks, and control animals were plotted for four different eccentricities from the optic nerve head (1 = closest to optic nerve head). Five and six weeks after immunizations, animals expressed a lower retinal ganglion cell density. (**C**) At four, five, and six weeks, mean antigen-antibody reactivity of the serum of HSP27 and control animals was plotted against the corresponding molecular weight of the retinal antigen and revealed, e.g., a significant up- and down-regulations of certain antibodies reactivities. Scale bar = 10 µm [46].

In a follow-up study, our group aimed to investigate the role of these autoantibodies in cell death in more detail. Systemic immunization with HSP27 resulted in a significant loss of retinal ganglion cells after five and six weeks (Figure 2A,B), while the intraocular pressure (IOP) remained stable in these animals [46]. A systemic immune response was noted after the HSP27 immunization, leading to altered IgG antibody patterns, with certain time-dependent up- and down-regulations (Figure 2C).

**Figure 3:**
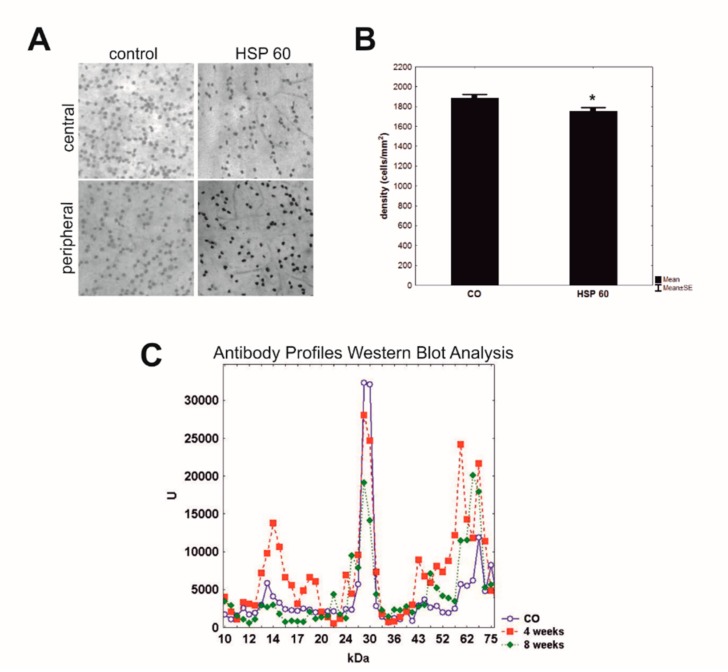
(**A**) Retinal ganglion cells were labeled by Brn3a in retinal flatmounts of rats immunized with HSP60 and controls. The central and peripheral part of retina (40x magnification) were analyzed. (**B**) After eight weeks, rats immunized with HSP60 showed a significant loss of retinal ganglion cells in comparison to control animals (CO; *p = 0.02). Values are mean ± SEM. (**C**) Mean antigen-antibody reactivity of the serum of the HSP60 group at four and eight weeks and control group (CO) was plotted against the corresponding molecular weight of the retinal antigen. Complex antibody regulations could be detected four and eight weeks after immunization with HSP60. These changes altered according to the time after immunization. [50].

Immunization of HSP27 in combination with glial cell line-derived neurotrophic factor or S100B, two factors for which autoantibodies were also detectable in glaucoma patients, did not cause additional damage or more severe retinal ganglion cell loss. Therefore, we assume that both antigens might interact, possibly having inhibitory effects on each other, and thus prevent additional damage to the retina [47,48,49]. The effect of HSP60 immunization was also investigated. A significant loss of retinal ganglion cells and their axons was noted in HSP60 immunized rats after eight weeks (Figure 3A,B), while an IOP elevation was not detectable. Additionally, complex antibody profiles were detected in serum samples four and eight weeks after immunization with HSP60 (Figure 3C). This IgG antibody pattern altered throughout the study. Elevated serum autoantibodies were noted, e.g., against HSP60, S-arrestin, and GFAP, but down-regulations were also observed [50]. 

Based on these results, autoantibodies to HSPs appear to play a significant role in the degeneration of the retina. In a follow-up project, it was investigated whether a local application of HSP27 could lead to similar glaucoma-like damage in animals. Hence, a high HSP27 concentration was applied intravitreally. This led to an IOP-independent loss of retinal ganglion and amacrine cells 21 days after the injection. Interestingly, first signs of neurofilament degeneration in the optic nerve were also detectable after 21 days, whereas glial changes could not be observed. Thus, direct high levels of non-phosphorylated extracellular HSP27 seem to induce glaucoma-like damage in these animals [51]. One potential mechanism for this damage is a HSP27-mediated initiation of the immune system by toll-like receptors (TLRs), or an increased ubiquitin-mediated degradation of the cAMP response element-binding protein, which is responsible for the reduction of inflammatory processes. Therefore, the question remains how precisely the injected HSP27 acts on the cells and contributes to the development of glaucomatous damage and is therefore part of further research.

The role of HSPs was not only studied in glaucoma models without elevated IOP. An increased immunostaining for HSP27, HSP60, and HSP90 was observed in retinae of monkeys with ocular hypertension (OHT) glaucoma, especially in the ganglion cell and nerve fiber layer [52]. The authors postulate that these HSPs are possibly up-regulated to inhibit apoptosis and rescue neuronal cells. Moreover, an IOP increase in a rat animal model led to an elevated HSP27 expression in the retina [53,54]. An up-regulation of HSP27 might be an internal cell protective mechanism and is considered a marker for neuronal injury. Additionally, in an established microbead OHT model, IOP elevation led to a degeneration of retinal ganglion cells as well as their axons, and induced infiltration of autoreactive CD4^+^ T-cells into the retina. These T-cells were pre-sensitized by the commensal microflora. In addition, an up-regulation of extracellular and membrane-bound HSP27 in the retinal ganglion cell layer could be observed. Based on these results, it was speculated whether HSP27-imprinted CD4^+^ T-cells penetrate the retina and thus cross-react with the HSP27 expressing cells of the retinal ganglion cell layer [55]. Chidlow et al. characterized the expression patterns of HSP27 and HSP70 in the retina and optic nerve in different models of retinal degeneration, namely optic nerve crush, NMDA-model, chronic hypoperfusion, and OHT. They noted an up-regulation of HSP27 in the retina in all these models. Also, optic nerves showed a HSP27 increase during optic nerve degeneration. HSP70, on the other hand, was not increased in these models [56]. Moreover, Wang et al. noted an elevated expression of HSP72 in the retina of an OHT glaucoma model via immunohistochemistry [57]. HSP72 expression is usually low, but is upregulated during stress or as a result of injury, to protect neuronal cells like retinal ganglion cells [58]. Furthermore, several studies analyzed the IOP-induced gene modulation of small heat shock proteins, the crystallins. They found a transient down-regulation of crystallins in different animals models, such as after trabecular laser photocoagulation [59], or injection of hypertonic saline into the episcleral vein [54], and in a hereditary rat model with elevated IOP [60]. Since crystallins are known for their anti-apoptotic and cytoprotective effects, and thus are up-regulated in response to stress and injury, their down-regulation after IOP elevation may be related to retinal ganglion cell death in glaucoma. Interestingly, the down-regulation of the genes was only transient, and was followed by an up-regulation during the phase of ongoing neurodegeneration [61].

Taken together, these results show that several HSPs could be involved in the development of glaucomatous damage.

## 5. Heat Shock Proteins: Friend or Foe

Findings from clinical and experimental studies hint towards an involvement of HSPs or HSP antibodies in glaucoma pathogenesis. Their exact role has not been conclusively clarified yet and seems to be either harmful or helpful, depending on the situation.

First and foremost, HSPs are known for their protective and helpful functions. As chaperones, they contribute to the refolding of denatured proteins and thus prevent additional damage [2]. Furthermore, α-crystallins, HSP27, and HSP70 are known regulators of apoptosis. As such, HSP27 can bind cytochrome C and prevent the formation of the apoptosome, as well as the concomitant mitochondrial pathway of caspase-dependent apoptosis [62]. Likewise, an increased HSP70 level in stressed cells leads to a reduction of caspase 3, and thus to a decreased apoptosis level [63]. The protective effects of crystallins are multifarious and based inter alia on their interaction with the proapoptotic regulators Bax/Bcl-X [64], and their inhibition of caspase 3 [65,66]. In addition to their apoptosis regulation capacity, HSPs also have a stabilizing effect on the cytoskeleton, which in turn results in cell protective effects [63]. Moreover, some HSPs, like crystallins, support retinal axon growth and repair, thereby offering interesting targets for regenerative treatments also for glaucoma [67].

Since misfolding of specific peptides is often involved in neurodegenerative diseases, certain HSPs could be used protectively in this course. This assumption is supported by the fact that surviving neuronal cells often overexpress HSPs. Thus, the increased expression of HSP70 in animal models of Parkinson’s [68] and Alzheimer’s disease [69,70] led to a delay in disease symptoms by blocking the function of several pro-apoptotic key factors [68,69,71]. Furthermore, in an ischemia-reperfusion injury animal model, beneficial effects on the retinal ganglion cells could be observed after electroporation of HSP27 in the vitreous [72]. Interestingly, the induction of HSP72 by heat shock or zinc administration in a rat model of acute glaucoma supported retinal ganglion cell survival [73,74,75]. Therefore, the authors assume that an enhanced induction of endogenous HSP72 could be a possible novel therapeutic approach for glaucoma patients. In addition, the intravitreal injection of αB-crystallin in an experimental animal model of glaucoma exhibited neuroprotective effects on morphological and functional level [76].

Only a few examples for clinical administrations of HSPs are described so far. Most applications can be found in rheumatoid arthritis. In a pilot trail, bacterial HSP dnaJ, also known as HSP40, was administered orally to patients with early rheumatoid arthritis. The result of this HSP40 application was an increased production of the regulatory cytokines interleukin (IL)-4 and IL-10, a decreased T-cell proliferation, and a reduction of the proinflammatory cytokines IL-2, interferon-γ, and tumor necrosis factor (TNF)-α. In addition, data collected by physicians and patients’ self-assessment for joint values and overall disease activity showed a reduction of the disease symptoms [77]. In another study, patients with early rheumatoid arthritis were treated intravenously with HSP10. In contrast to HSP27, HSP10 has anti-inflammatory properties by inhibiting the NF-κB mediated signaling pathway. The HSP10 therapy significantly reduced disease symptoms [78]. 

Nevertheless, there is also another side to HSPs. It is known that high extracellular HSP concentrations are recognized as danger signals and subsequently activate the immune system. This occurs on the one hand via binding to TLRs, such as TLR-2 and TLR-4 [79]. As a result of the HSP-TRL binding, the transcription factor NF-κB is phosphorylated, leading to an increased release of proinflammatory cytokines, such as IL-6 and IL-8 [80]. On the other hand, as mentioned before, HSPs exhibit a highly conserved structure, which leads to cross- and autoimmune reactions. Especially T-cells directed against the bacterial HSP could cross-react with endogenous homologous HSP due to molecular mimicry and induce thus autoimmune damage [81]. In line with this, HSP60 is a target of autoreactive T-cells in chronic inflammation and arteriosclerosis [82]. Recently, in a microbead OHT glaucoma model, the importance of cross-reactive T-cells was investigated. The authors concluded that HSP27-imprinted CD4^+^ T-cells penetrate the retina, thus cross-react with the HSP27 expressing cells of the retinal ganglion cell layer and induce their cell death [55]. Furthermore, HSP70 can stimulate the production of proinflammatory cytokines such as IL-1, IL-6, and TNF-α via the CD14-dependent signaling pathway to promote cell damage [79]. Collectively, these data suggest that HSPs may be directly involved in inflammatory processes and increase inflammatory damage [71]. For this reason, HSPs appear to be up-regulated in the early stages of neurodegenerative diseases, such as glaucoma, due to their neuroprotective functions. However, a prolonged up-regulation of HSP expression and its evoked immune response can lead to increased or additional cell death. Therefore, HSPs seem to be friend and foe in glaucoma disease.

## 6. Conclusions

The exact mechanisms leading to glaucoma, one of the most common causes of blindness, are still unknown. The immune system and the interaction with HSPs seem to be key player.

HSPs, like HSP27 or HSP60, exhibit neuroprotective properties in the central nervous system due to their chaperoning and anti-apoptotic activities. Therefore, up-regulation of HSPs in the early stages of glaucoma seems to be neuroprotective and a possible novel therapeutic approach for patients. Nevertheless, in an animal model, immunization with HSPs induced pressure-independent retinal ganglion cell degeneration and axon loss, resulting in glaucoma-like damage. Moreover, the development of autoreactive antibodies as well as glial and T-cell activation has been described in this model. Recently, we found that intravitreal HSP27 injection also causes degeneration of retinal ganglion cells and their axons. Also, in a pressure-dependent glaucoma animal model, the infiltration of autoreactive T-cells in the retina could be observed, and HSP were identified as target antigens of T-cell response. Thus, HSPs appear to have a direct damaging effect on retinal cells and seems to be playmakers in glaucoma pathogenesis.

For this reason, HSPs appear to be friend and foe in glaucoma pathogenesis depending on amount, induction-time, induction-region, cell type, and HSP type. The various functions of HSPs and the apparent changes in function should be further investigated, before HSPs can be used as therapeutic approach for glaucoma patients.
